# Higher Carbohydrate Antigen 125 Levels Are Associated with Increased Risk of Coronary Heart Disease in Elderly Chinese: A Population-Based Case-Control Study

**DOI:** 10.1371/journal.pone.0081328

**Published:** 2013-11-26

**Authors:** Xiaorong Li, Meian He, Jiang Zhu, Ping Yao, Xiulou Li, Jing Yuan, Xinwen Min, Mingjian Lang, Handong Yang, Frank B. Hu, Tangchun Wu, Sheng Wei

**Affiliations:** 1 Department of Epidemiology and Biostatistics, School of Public Health, Tongji Medical College of Huazhong University of Science and Technology, Wuhan, China; 2 MOE Key Lab of Environment and Health, School of Public Health, Tongji Medical College, Huazhong University of Science & Technology, Wuhan, China; 3 Dongfeng Central Hospital, Dongfeng Motor Corporation and Hubei University of Medicine, Shiyan, Hubei, China; 4 Departments of Nutrition and Epidemiology, Harvard School of Public Health, Boston, Massachusetts, United States of America; Brigham & Women's Hospital, and Harvard Medical School, United States of America

## Abstract

**Background:**

High carbohydrate antigen 125 (CA-125) level was reported to be associated with some cardiac dysfunctions, such as chronic heart failure, but the relationship between CA-125 level and coronary heart disease (CHD) risk remains unclear. The aim of this study was to explore the potential association in a Chinese older population.

**Methods:**

In a population-based case-control study conducted in a Chinese older population, serum CA-125 levels were measured in 1177 diagnosed CHD patients and 3531 age and sex matched control subjects without CHD.

**Results:**

Serum CA-125 level was significantly higher in CHD patients than controls (*P* < 0.001) with adjustment for age, gender, smoking, drinking, BMI, physical activity, hypertension, dyslipidemia, diabetes mellitus, medication history and family history of CHD and myocardial infarction. CHD risk was doubled (OR: 2.10, 95%CI: 1.69-2.60) among subjects in the highest quartile compared to those in the lowest quartile of CA-125 level (*P*_trend_ < 0.001). Furthermore, CA-125 levels were associated with CHD risks in subjects with age over 60 years (OR: 2.19, 95%CI: 1.75-2.73), current smokers (OR: 2.29, 95%CI: 1.50-3.49), current drinkers (OR: 2.35, 95%CI: 1.57-3.53) and subjects with hypertension (OR: 2.04, 95%CI: 1.71-2.43).

**Conclusions:**

Elevated serum CA-125 level might be associated with increased risk of coronary heart disease in the Chinese older population. Further investigations are needed to identify the possible biological role of CA-125 in CHD development in the future.

## Introduction

CA-125 (Carbohydrate antigen 125) is a well-established tumor marker to monitor the efficacy of ovarian cancer therapy and early detect its recurrence [1]. But it is also elevated in both ovarian and non-ovarian diseases, malignant and non-malignant conditions [2]. Interestingly, there are evidences which showed that, CA-125 could be secreted from mesothelial cells as well as tumoural origin tissue [3,4]. Recent studies have also demonstrated that serum CA-125 level was significantly elevated in chronic heart failure patients [5-9]. Furthermore, increasing evidences have indicated that high serum CA-125 levels were associated with risk of hypertrophic cardiomyopathy, pseudoaneurysm of the left ventricular lateral wall, cardiac angiosarcoma, pericardial tamponade, infective perimyocarditis and atrial fibrillation [10-15]. However, few studies have investigated whether the higher concentration of serum CA-125 is associated with the increased risk of coronary heart disease (CHD) or not until now.

Coronary heart disease (CHD) is a condition in which there is an inadequate supply of blood and oxygen to a portion of the myocardium; it typically occurs when there is an imbalance between myocardial oxygen supply and demand. It is one of the leading causes of mortality and disability in both industrialized and developing countries [16-18]. It was estimated that heart disease and stroke are projected to be the single leading cause of death by 2030 [19]. CHD is a multi-factorial disease and the underlying mechanisms of CHD have not been well elucidated clearly. Inflammation is considered to play an important role in the development of atherosclerosis [20,21]. Systemic inflammation could enhance atherogenesis and inflammatory components could drive the formation, progression and rupture of atherosclerotic plaques for inflammation could promote loss of endothelium, the hallmark of superficial erosion [22-25]. Previous studies have also found that CA-125 could be produced by mesothelial cells as a consequence of inflammation, stasis or other stimulatory mechanisms in patients with heart failure [5,6,9,26]. Recently, a small case-control study also suggested inflammation and cytokine levels may be responsible for CA-125 production and release [27]. Given this evidence, it is reasonable to hypothesize that there may be a potential association between serum CA-125 level and CHD risk.

Here, we performed a large population-based case-control study to explore the association between serum CA-125 level and the CHD risk in Chinese older population, which was based on Dongfeng-Tongji cohort study of retired workers in Shiyan, Hubei province, China. 

## Methods

### Ethics statement

Ethical approval was obtained from the Medical Ethics Committee of the School of Public Health, Tongji Medical College, Huazhong University of Science and Technology.

### Study population and design

We used data from Dongfeng-Tongji cohort (DFTJ cohort) study of retired workers, which was described in detail elsewhere [28]. Briefly, this cohort was launched in 2008 among retirees of Dongfeng Motor Corporation (DMC) in Shiyan City, Hubei province, China. For the current study, we included 1177 documented CHD cases and 3531frequency-matched (by age and gender) controls between September 2008 and June 2010. CHD was confirmed on the basis of the most recent international guidelines: a combination of classical symptoms with positive results from 12-lead electrocardiograph (ECG), cardiac enzymes, functional or stress test, or coronary angiography using standard Judkins techniques (significant coronary artery stenoses≥50% in at least one major coronary artery) [29]. A total of 55.73% (656) CHD cases underwent coronary angiography. Nonfatal myocardial infarction was diagnosed by a development of pathologic Q waves on serial ECG, elevations of cardiac enzyme values, or medical records for clinical symptoms and signs. 210 of CHD cases were diagnosed as MI according to their medical record.

Controls were randomly selected from retired employees employed in the same DFTJ cohort on the basis of medical history, clinical examinations, electrocardiography and face-to-face inquiry at admission. None of them had abnormalities of ECG or diagnostic evidence of CHD. Hypertension was defined as systolic/ diastolic blood pressure≥140/90mmHg in at least two measurements, or current use of anti-hypertensive medicine for the most recent 2 weeks, or a previous diagnosis of hypertension by a clinician. Diabetes mellitus (DM) was defined by the WHO criteria [30]: fasting blood glucose (FBG)≥7mmol/L or a 2 hour postprandial plasma glucose ≥11.1mmol/L, or a prescription history of antidiabetic medications, including oral antidiabetics, incretin products, and insulin during the most previous two weeks, or a previous diagnosis of DM. Dyslipidemia was diagnosed as total cholesterol (TC) concentration≥5.72 mmol/L or triglyceride (TG) concentration ≥1.70 mmol/L or high-density lipoprotein cholesterol (HDL-C) concentration≤0.91mmol/L, or use medicine of dyslipidemia during the previous two weeks, or a positive history for dyslipidemia. All chronic diseases were verified through medical record reviews.

Subjects with a history of congenital heart disease, chronic heart failure, recent acute coronary syndrome (< 6 months), vascular disease, valvular disease, cardiomyopathy and neoplastic diseases and cancers were excluded through medical record review based on clinical symptoms, physical examination, electrocardiogram and chest X-ray.

After obtained written informed consent from every participant, a semi-structural questionnaire was used to collect baseline information by trained interviewers during face-to-face interviews. The medical examination was performed at the same time. Demographic information, socio-economic status, family and personal disease histories, medication history in recent two weeks, alcohol use and smoking consumption were also inquired in the questionnaire.

Educational levels were categorized as low (primary school or illiteracy), medium (junior high school) and high (senior high school, university or college or higher). Body mass index (BMI) was calculated as weight in kilograms divided by height in meters squared. 

### Laboratory measurement

After an overnight fast, five milliliters of fasting blood was drawn from all subjects with a vacuum coagulation tube for serum in the morning. We measured the serum CA-125 level with the ARCHITECT Ci8200 automatic analyzer (ABBOTT Laboratories. Abbott Park, Illinois, U.S.A.) using the Abbott Diagnostics reagents according to the manufacturer’s instructions at Dongfeng Central Hospital’s laboratory. The method used by the ARCITECT Ci8200 platform for serum CA-125 levels measurement is microparticle chemiluminescent immunoassay (CMIA).The intra-assay coefficients of variation were 5.50% for CA-125. Blood glucose and blood lipids (including TC, TG and HDL-C) measurements were described in our previous study [28].

### Statistical analysis

Characteristics of study subjects are presented as mean (SD) for continuous variables and as percentages for categorical data. All continuous variables were tested for normal distribution with the Kolmogorov-Smirnov test. For serum CA-125 levels, the cut off values for division of data into concentration categories were based on the distribution in controls. Continuous variables were analyzed by 2-tailed t tests for normal distributions and the Mann-Whitney U test for nonparametric distributions. Categorical data were evaluated by Chi-square test as appropriate. Odds ratios and 95% confident intervals were estimated by logistic regression with and without adjustment for age, gender, smoking, drinking, BMI, physical activity, hypertension, dyslipidemia, diabetes mellitus, medication history and family history of CHD and myocardial infarction. All tests were two-sided and α < 0.05 was considered statistically significant. Analyses were performed using SPSS software (version 12.0; SPSS Inc., Chicago, IL, USA).

## Results

### Characteristics of study population

The demographic characteristics of the study population are presented in Table 1. When compared with the controls, smoking (*P* < 0.001) and higher BMI (*P* < 0.001) were more common in cases while lower DBP (*P* < 0.001) and less drinking (*P* < 0.001) were found among cases. Total cholesterol (TC) and high-density lipoprotein cholesterol (HDL-C) were lower in CHD cases than controls (P < 0.001). As expected, CHD patients were more likely to have hypertension, dyslipidemia and diabetes mellitus (*P* < 0.001, respectively). Pharmacological drugs (antibiotics, anticoagulant, anti-hypertensive and hypoglycemic drugs) were more often used in cases (*P* < 0.001, respectively). Median and range of CA-125 level were 7.60 U/ml (3.50-11.40 U/ml) in cases and 5.54 U/ml (1.31-9.90 U/ml) in controls. Serum CA-125 level was significantly higher in CHD cases than that in controls (*P* < 0.001).

**Table 1:** Demographic characteristics of study subjects.

**Demographic and risk factors**		**Cases (n = 1177)**	**Controls (n = 3531)**	***P*-value**
**Age**				0.995
< 60		106 (9.01)	319 (9.03)	
60-		566 (48.09)	1703 (48.23)	
≥ 70		505 (42.91)	1509 (42.74)	
**Gender**				0.93
Male		616 (52.34)	1853 (52.48)	
Female		561 (47.66)	1678 (47.52)	
**Education levels^†^**				0.65
Low		395 (33.85)	1149 (32.79)	
Medium		402(34.45)	1194 (34.08)	
High		370 (31.71)	1161 (33.13)	
**BMI (kg / m^2^)**				< 0.001
< 23.9		371 (32.04)	1605 (46.44)	
24-		552 (47.67)	1387 (40.13)	
≥ 28		235 (20.29)	464 (13.43)	
**Physical activity**		1043 (88.62)	3198 (90.57)	0.052
**Blood pressure(mmHg)**				
SBP		131.91 ± 17.36	131.29 ± 18.25	0.31
DBP		76.15 ± 10.47	77.35 ± 10.47	0.001
**TC(mmol/L)**		4.89 ± 1.14	5.21 ± 0.97	< 0.001
**TG (mmol/L)**		1.49 ± 0.95	1.43 ± 1.05	0.12
**HDL (mmol/L)**		1.34 ± 0.36	1.44 ± 0.41	< 0.001
**Smoking status**				< 0.001
Current		148 (12.60)	693 (19.73)	
Former		277 (23.57)	415 (11.81)	
Never		750 (63.83)	2405 (68.46)	
**Drinking status**				< 0.001
Current		170 (14.46)	788 (22.32)	
Former		133 (11.31)	159 (4.50)	
Never		873 (74.23)	2583 (73.17)	
**Medication history**				
Antibiotics	114 (9.69)		177 (5.01)	< 0.001
Anticoagulant	682 (57.94)		359 (10.17)	< 0.001
Anti-hypertensive medication	770 (65.42)		965 (27.33)	< 0.001
Hypoglycemic medication	257 (21.84)		346 (9.80)	< 0.001
**Disease History**				
Hypertension		942 (80.03)	1919 (54.35)	< 0.001
Diabetes mellitus		371 (31.52)	664 (18.80)	< 0.001
Dyslipidemia		905 (76.89)	1932 (54.72)	< 0.001
**Family history of disease**				
Coronary heart disease		183 (15.55)	186 (5.27)	< 0.001
Myocardial infarction		38 (3.29)	32 (0.92)	< 0.001
**CA-125 level (U/ml)**		7.60 (3.50-11.40)	5.54 (1.31-9.90)	< 0.001^*^

Data are presented as number (percentage) or mean ± SD unless noted otherwise. CA-125 is presented as median (25th- 75th quartile). *P* values were calculated using 2-tailed t tests or Chi-square test.

*P* ^*^value was obtained using the Mann-Whitney U test.

Education levels**^†^**: Low, Primary school or illiteracy; Medium, Junior high school; High, Senior high school, university or college or higher.

Abbreviations: TC, total cholesterol; TG, triglyceride; HDL, high-density lipoprotein.

### Serum CA-125 level and CHD risk

Analyses of CA-125 level in quartiles (Table 2) showed that the risk of CHD was doubled (OR: 2.10, 95%CI: 1.69-2.60) in the highest quartile of CA-125 level (CA-125≥9.90U/ml) compared to those in the lowest quartile (CA-125≤1.31U/ml). The risk among those in the third quartile (OR: 2.08) of CA-125 levels was similar to those in the fourth quartile (OR: 2.10). These two groups therefore were combined into a group as “high” CA-125 level and similarly, the first and the second quartiles were combined into the “low” CA-125 level group for further stratified analyses.

**Table 2:** The association between CA-125 level and CHD risk in Chinese older population.

**Quartile**	**CA-125 level (U/ml)**	**Cases, n (%)**	**Controls, n (%)**	**OR (95% CI)**	**OR^‡^ (95% CI)**
First	≤ 1.31	191 (16.23)	881 (24.95)	1.00	1.00
Second	1.31-5.54	215 (18.27)	884 (25.04)	1.12 (0.90-1.39)	1.11 (0.87-1.40)
Third	5.54-9.90	380 (32.29)	877 (24.84)	2.00 (1.64-2.44)	2.08 (1.67-2.58)
Fourth	≥ 9.90	391 (33.22)	889 (25.18)	2.03 (1.67-2.47)	2.10 (1.69-2.60)
				*P*_trend_< 0.001	*P*_trend_< 0.001

OR: Crude OR. OR^‡^: Adjusted for age, gender, smoking, drinking, BMI, physical activity, hypertension, dyslipidemia, diabetes mellitus, medication history and family history of CHD and myocardial infarction.

As shown in Table 3, the association between CA-125 level and CHD risk tended to be stronger among older persons (OR: 2.19, 95%CI: 1.75-2.73), males (OR: 2.07, 95%CI: 1.67-2.56) and overweight (OR: 2.03, 95%CI: 1.62-2.55) after adjustment for age, gender, smoking, drinking, BMI, physical activity, hypertension, dyslipidemia, diabetes mellitus, medication history and family history of CHD and myocardial infarction. The CHD risk was inclined to be stronger among current smokers (OR: 2.29, 95%CI: 1.50-3.49), current drinkers (OR: 2.35, 95%CI: 1.57-3.53) and persons with hypertension (OR: 2.04, 95%CI: 1.71-2.43), without diabetes mellitus (OR: 2.09, 95%CI: 1.75-2.50) and dyslipidemia (OR: 2.21, 95%CI: 1.66-2.95).

**Table 3:** Associations between CA-125 level and CHD risk by subgroups.

**Risk factors**	**Level^§^**		**Cases**	**Controls**	**Crude OR (95% CI)**	***OR*^#^ (95% CI)**
**Age**						
< 60		Low	51	163	1.00	1.00
		High	55	156	1.13 (0.73-1.75)	1.44 (0.83-2.51)
60-		Low	201	895	1.00	1.00
		High	365	808	2.01 (1.65-2.45)	2.19 (1.75-2.73)
≥ 70		Low	154	707	1.00	1.00
		High	351	802	2.01 (1.62-2.49)	2.00 (1.58-2.53)
**Gender**						
Male		Low	198	898	1.00	1.00
		High	418	955	1.99 (1.64-2.41)	2.07 (1.67-2.56)
Female		Low	208	867	1.00	1.00
		High	353	811	1.81 (1.49-2.21)	1.96 (1.58-2.44)
**BMI**						
< 23.9		Low	127	807	1.00	1.00
		High	244	798	1.94 (1.54-2.46)	2.00 (1.55-2.58)
24-		Low	189	692	1.00	1.00
		High	363	695	1.91 (1.56-2.35)	2.03 (1.62-2.55)
≥ 28		Low	87	246	1.00	1.00
		High	156	245	1.80 (1.31-2.47)	1.93 (1.34-2.78)
**Smoking status**						
Current		Low	50	360	1.00	1.00
		High	98	333	2.12 (1.46-3.07)	2.29 (1.50-3.49)
Former		Low	90	188	1.00	1.00
		High	187	227	1.72 (1.25-2.36)	1.93 (1.34-2.77)
Never		Low	264	1207	1.00	1.00
		High	486	1198	1.86 (1.57-2.20)	1.93 (1.60-2.33)
**Drinking status**						
Current		Low	49	399	1.00	1.00
		High	121	389	2.53 (1.77-3.63)	2.35 (1.57-3.53)
Former		Low	51	63	1.00	1.00
		High	82	96	1.06 (0.66-1.69)	1.21 (0.69-2.12)
Never		Low	305	1303	1.00	1.00
		High	568	1280	1.90 (1.62-2.22)	2.05 (1.72-2.45)
**Disease history**						
**Hypertension**							
Yes		Low	330	1009	1.00	1.00	
		High	612	910	2.06 (1.75-2.42)	2.04 (1.71-2.43)	
No		Low	76	756	1.00	1.00	
		High	159	856	1.85 (1.38-2.47)	1.91 (1.41-2.60)	
**Dyslipidemia**							
Yes		Low	314	958	1.00	1.00	
		High	591	974	1.85 (1.57-2.18)	1.91 (1.59-2.28)	
No		Low	92	807	1.00	1.00	
		High	180	792	1.99 (1.52-2.61)	2.21 (1.66-2.95)	
**Diabetes mellitus**							
Yes		Low	123	284	1.00	1.00	
		High	248	380	1.51 (1.16-1.97)	1.59 (1.17-2.15)	
No		Low	283	1481	1.00	1.00	
		High	523	1386	1.98 (1.68-2.32)	2.09 (1.75-2.50)	

Low**^§^**: the first and second quartiles; High: the third and fourth quartiles.

OR**^#^**: Adjusted for age, gender, smoking, drinking, BMI, physical activity, hypertension, dyslipidemia, diabetes mellitus, medication history and family history of CHD and myocardial infarction.

We further analyzed serum CA-125 level and myocardial infarction (MI) risk in our population (Table S1), of the 210 CHD patients having MI, higher level of CA-125 was related to higher MI risk in a dose-responsive manner with adjustment for age, gender, smoking, drinking, BMI, physical activity, hypertension, dyslipidemia, diabetes mellitus, medication history and family history of CHD and myocardial infarction.

### CA-125 level and the severity of coronary artery stenoses in CHD

 We divided the 1177 CHD cases into two groups: CHD accompanying with MI (210, Table S1) and CHD without MI (967, Table S2), and then compared the two groups with controls (3531) respectively, the adjusted OR of CHD patients accompanying with MI in highest quartile of CA-125 level was 2.76 (95%CI: 1.73-4.41, *P* < 0.001), and that of CHD patients without MI was 2.02 (95%CI: 1.61-2.54, *P* < 0.001) after adjustment for age, gender, smoking, drinking, BMI, physical activity, hypertension, dyslipidemia, diabetes mellitus, medication history and family history of CHD and myocardial infarction.

### Relation of CHD risk factors and serum CA-125 level among controls

Serum CA-125 level of controls ascended substantially with increasing age (*P*_*trend*_ < 0.001; Table 4). Males tended to have higher serum CA-125 level than females (*P* = 0.015). Smokers had significantly higher CA-125 level than nonsmokers (*P* = 0.04). Controls with hypertension have significantly lower serum CA-125 level than those without hypertension (*P* = 0.001). Contrary results were found in controls with and without diabetes mellitus (*P* < 0.001). Controls taken hypoglycemic drugs tend to have significantly higher level of CA-125 (*P* = 0.03). 

**Table 4:** Relation between CHD risk factors and CA-125 level among controls.

**Demographic and risk factors**	**N (n = 3531)**	**CA-125, mean ± SD (U/ml)**	***P*-value**
**Age**			0.001
< 60	319	6.90 ± 8.34	
60-	1703	6.25 ± 6.99	
≥ 70	1509	7.04 ± 6.64	
**Gender**			0.01
Male	1853	6.96 ± 7.69	
Female	1678	6.30 ± 6.11	
**BMI**			0.07
< 23.9	1605	6.55 ± 7.00	
24-	1387	6.65 ± 7.29	
≥ 28	464	7.03 ± 6.20	
**Medication history**			
Antibiotics			0.93
Yes	177	6.74 ± 6.96	
No	3354	6.64 ± 6.99	
Anticoagulant			0.07
Yes	359	7.10 ± 6.21	
No	3172	6.60 ± 7.07	
Anti-hypertensive medication			0.31
Yes	965	6.68 ± 6.00	
No	2566	6.63 ± 7.33	
Hypoglycemic medication			0.03
Yes	346	7.17 ± 6.08	
No	3185	6.59 ± 7.08	
**Smoking status**			0.04
Current	693	6.69 ± 7.54	
Former	415	7.62 ± 9.84	
Never	2405	6.47 ± 6.19	
**Drinking status**			0.11
Current	788	6.80 ± 7.24	
Former	159	8.37 ± 13.75	
Never	2583	6.49 ± 6.24	
**Disease History**			
Hypertension			0.001
Yes	1919	6.36 ± 6.44	
No	1612	6.99 ± 7.57	
Dyslipidemia			> 0.05
Yes	1932	6.84 ± 7.41	
No	1599	6.40 ± 6.44	
Diabetes mellitus			< 0.001
Yes	664	7.41 ± 6.26	
No	2867	6.47 ± 7.14	

## Discussion

In this population-based case-control study, we investigated the association between serum CA-125 level and the risk of CHD in a large Chinese older population for the first time. We observed that the elevated serum CA-125 level was associated with a higher risk of CHD, and such associations were also evident in older individuals, males, current smokers and drinkers, overweight individuals, and those who had hypertension.

In early time, serum CA-125 was found to be related to diastolic and systolic parameters, ejection fraction and myocardial performance index in patients with chronic HF [31]. Subsequently, elevated CA-125 level has also been observed in other cardiac pathologies such as aortic stenosis, mitral stenosis, mitral valve endocarditis, acute myocardial infarction [32-36]. However, in stark contrast, little data has been published concerning the association of the concentration of serum CA-125 and CHD risk until now. 

As to the possible mechanisms involved in increased CA-125 level observed in HF, De Gennaro et al. suggested that haemodynamic abnormalities and inflammatory cytokines may play significant roles in the development of atherosclerosis and its complications [37]. A few later studies have also demonstrated that CA-125 is expressed in different tissues derived from coelomic epithelium in response to various stimuli, including mechanical stress and inflammatory stimuli [38-41]. Furthermore, under normal circumstances, mesothelial cells could maintain a steady-state with proliferation balanced by cell death. However, such balance is disrupted when mesothelial cells are exposed to mechanical stress and inflammatory stimuli in the early phase of atherosclerosis. On the one hand, mesothelial cells might synthesize more hyaluronan and cytoplasmic fibers to defense the cellular injury and death [42,43]. Vitro experiments have demonstrated that the secretion of CA-125 could be enhanced by the inflammatory cytokines [44]. So inflammatory may be responsible for serum CA-125 role in CHD development. Our findings suggested that serum CA-125 was related to some inflammatory related status, such as smoking, hypertension and diabetes mellitus, may act as an evidence to support this hypothesis.

In our study, CA-125 level-associated CHD risks were higher in subjects aged over 60 years, overweight individuals, cigarette smoke and hypertension. The similar results have been found in the PLCO (Prostate, Lung, Colorectal and Ovarian Cancer) screening trial [45] except for subjects with hypertension. These factors may damage the endothelium and lead to the subsequent inflammatory reactions in the vascular wall, which in turn increases the production of primary proinflammatory cytokines [46]. The association of higher CA-125 level with higher CHD risk in older age could possibly be a consequence of aging processes at the cellular and immunological level [45]. Cigarette smoke is a major risk factor for CHD and produces a chronic inflammatory state that contributes to the atherogenic disease processes and elevates levels of biomarkers of inflammation. Besides, this chronic state of inflammation might be directly related to subsequent elevated risk for cardiovascular diseases and has detrimental effects on the metabolism and function of innate immune cells [47,48]. The plausibility is supported by our observation that among controls smokers tended to have higher level of serum CA-125 than nonsmokers.

An inverse correlation between CA-125 level and metabolic syndrome was recently reported in a study including 12,196 healthy Korean women [49]. Similar results have been also found in the present study. For example, controls with hypertension had significantly lower serum CA-125 level than those without hypertension. While conflicting findings showed that serum CA-125 levels were associated with lower risks of CHD in patients with dyslipdemia and diabetes mellitus after adjustment with confounding factors. That may be due to the different doses and/or the duration of the medicines they taken. However, the precise mechanisms should be investigated in future prospective studies.

In terms of limitations, the current case-control design limits the causal interpretation of the relationship between serum CA-125 level and the risk of coronary heart disease because the blood samples were collected from subjects having CHD events. Another limitation is that some CHD cases took multiple medications, which acted as a confounding factor when we analyzed the relationship between CA-125 level and CHD risk by subgroups. Thus, such findings need to be validated by future large prospective investigations. Furthermore, the role of unmeasured or residual confounding could not be ignored although we have adjusted for a wide range of CHD risk factors. Nevertheless, this is the first study to examine the association between serum CA-125 level and the risk of CHD in a large Chinese older population. Secondly, the sample size is considerable, which allows us to investigate the association between increased CA-125 level and CHD risk.

## Conclusions

In summary, our findings suggested that higher serum CA-125 level might be associated with a significantly increased risk of CHD in Chinese older population. Future prospective studies should be motivated by this finding to explore the precise mechanisms.

## Supporting Information

Table S1 **The association between CA-125 level and CHD with nonfatal MI in Chinese older population.**
(DOCX)

Table S2 **The association between CA-125 level and CHD without nonfatal MI in Chinese older population.**
(DOCX)
