# Supplementary material for: Higher Carbohydrate Antigen 125 Levels Are Associated with Increased Risk of Coronary Heart Disease in Elderly Chinese: A Population-Based Case-Control Study
Source: PLoS One. 2013 Nov 26;8(11):e81328. doi: 10.1371/journal.pone.0081328 (PMC3841119; doi:10.1371/journal.pone.0081328)
Supplement: Table S2 — The association between CA-125 level and CHD without nonfatal MI in Chinese older population. (DOCX) [file pone.0081328.s002.docx]

| **Table S2.**The association between CA-125 level and CHD without nonfatal MI in Chinese older population | | | | | |
| --- | --- | --- | --- | --- | --- |
| **Quartile** | **CA-125 level (U/ml)** | **Cases, n (%)** | **Controls, n (%)** | **OR (95% CI)** | **OR**^*^ **(95% CI)** |
| First | ≤ 1.31 | 156 (16.34) | 881 (24.95) | 1.00 | 1.00 |
| Second | 1.31-5.54 | 174 (18.22) | 884 (25.04) | 1.11 (0.88-1.41) | 1.10 (0.85-1.41) |
| Third | 5.54-9.90 | 321 (33.61) | 877 (24.84) | 2.07 (1.67-2.56) | 2.13 (1.70-2.69) |
| Fourth | ≥ 9.90 | 304 (31.83) | 889 (25.18) | 1.93 (1.56-2.39) | 2.02 (1.61-2.54) |
|  |  |  |  | *P*_trend_< 0.001 | *P*_trend_< 0.001 |
| OR: Crude OR.  OR^*^: Adjusted for age, gender, smoking, drinking, BMI, physical activity, hypertension, dyslipidemia, diabetes mellitus, medication history and family history of CHD and myocardial infarction. | | | | | |
